# Draft Genome Sequences of Multidrug-Resistant *Shigella* Strains Isolated from Diarrheal Patients in Bangladesh

**DOI:** 10.1128/MRA.00854-21

**Published:** 2021-10-21

**Authors:** Asaduzzaman Asad, Shoma Hayat, Fahmida Habib Nabila, Ruma Begum, Suraia Nusrin, Zhahirul Islam

**Affiliations:** a Laboratory of Gut-Brain Signaling, Laboratory Sciences and Services Division (LSSD), icddr,b, Dhaka, Bangladesh; b Department of Genetic Engineering and Biotechnology, East West University, Dhaka, Bangladesh; Montana State University

## Abstract

The emergence of multidrug-resistant (MDR) *Shigella* strains has impaired the efficacy of first-line antimicrobials and exacerbated diarrhea-associated morbidity and mortality worldwide. We report the draft genome sequences of 11 MDR *Shigella* strains isolated from the stool specimens of diarrheal patients in Bangladesh.

## ANNOUNCEMENT

Antimicrobial resistance (AMR) in *Shigella* spp. is a raging threat controlling feco-orally transmitted bacillary dysentery, or “shigellosis,” which causes the deaths of 40,000 children under 5 years annually (1, 2). The rapid spread of multidrug-resistant (MDR) *Shigella* spp. inactivates effective antibiotics and limits the shigellosis treatment options (2, 3). Moreover, the horizontal transfer of resistance factors through conjugative plasmids facilitates the acquisition of MDR in *Shigella* spp. and worsens the scenario. Thus, a clear understanding of the genetic modifications in MDR *Shigella* spp. is warranted in order to develop new treatment approaches (4).

*Shigella* strains were isolated from patients attending the treatment center operated by the International Centre for Diarrhoeal Disease Research, Bangladesh (icddr,b), in Dhaka, Bangladesh, using standard microbiological and biochemical methods (5). The isolated strains were cultured on MacConkey agar (Difco) aerobically for 18 h at 37°C and serologically confirmed by slide agglutination tests applying commercially available antiserum kits (Denka Seiken, Tokyo, Japan) (6). Susceptibility tests were performed on Mueller-Hinton agar after overnight growth at 37°C using antibiotic disks (Oxoid Ltd., England) and Epsilometer-test strips (AB-Biodisc, Solna, Sweden) as per the CLSI guidelines (7). The *Shigella* isolates were enriched in Luria-Bertani broth, and genomic DNA was extracted using the QIAamp DNA minikit (Qiagen). The quality and quantity of the DNA were checked using the NanoDrop spectrophotometer (Thermo Fisher Scientific, USA) and Qubit 2.0 fluorimeter (Life Technologies), respectively. The Illumina Nextera XT DNA library preparation kit was applied to construct a sequencing library from 1 ng genomic DNA. Sequencing was performed on the Illumina MiSeq platform using the Illumina MiSeq v3 reagent kit to generate 300-bp paired-end sequence reads. FastQC v0.11.9 was used for sequence quality checking, and Trimmomatic v0.36 was used for adapter trimming (8, 9). Additional trimming was performed using fastp v0.20.0, incorporating the “--corrction” flag (10). The resulting reads were *de novo* assembled using SPAdes v3.14.1, adding the “–careful” option (11). The assembled sequences were assessed using QUAST v5.0.2, and contigs shorter than 200 bp were discarded (12). Annotation was performed using the NCBI Prokaryotic Genome Annotation Pipeline (PGAP) v5.0 (13). The AMR and virulence genotypes were obtained using AMRFinderPlus v3.10.5 (14). Plasmids were identified using PlasmidFinder v2.1 (https://cge.cbs.dtu.dk/services/PlasmidFinder/), maintaining 90% threshold identity and 60% minimum coverage (15). Default parameters were used for all software unless otherwise specified. This study (PR-19048) was reviewed and approved by the institutional review board (IRB) and ethics committee of the icddr,b, Bangladesh.

**FIG 1:**
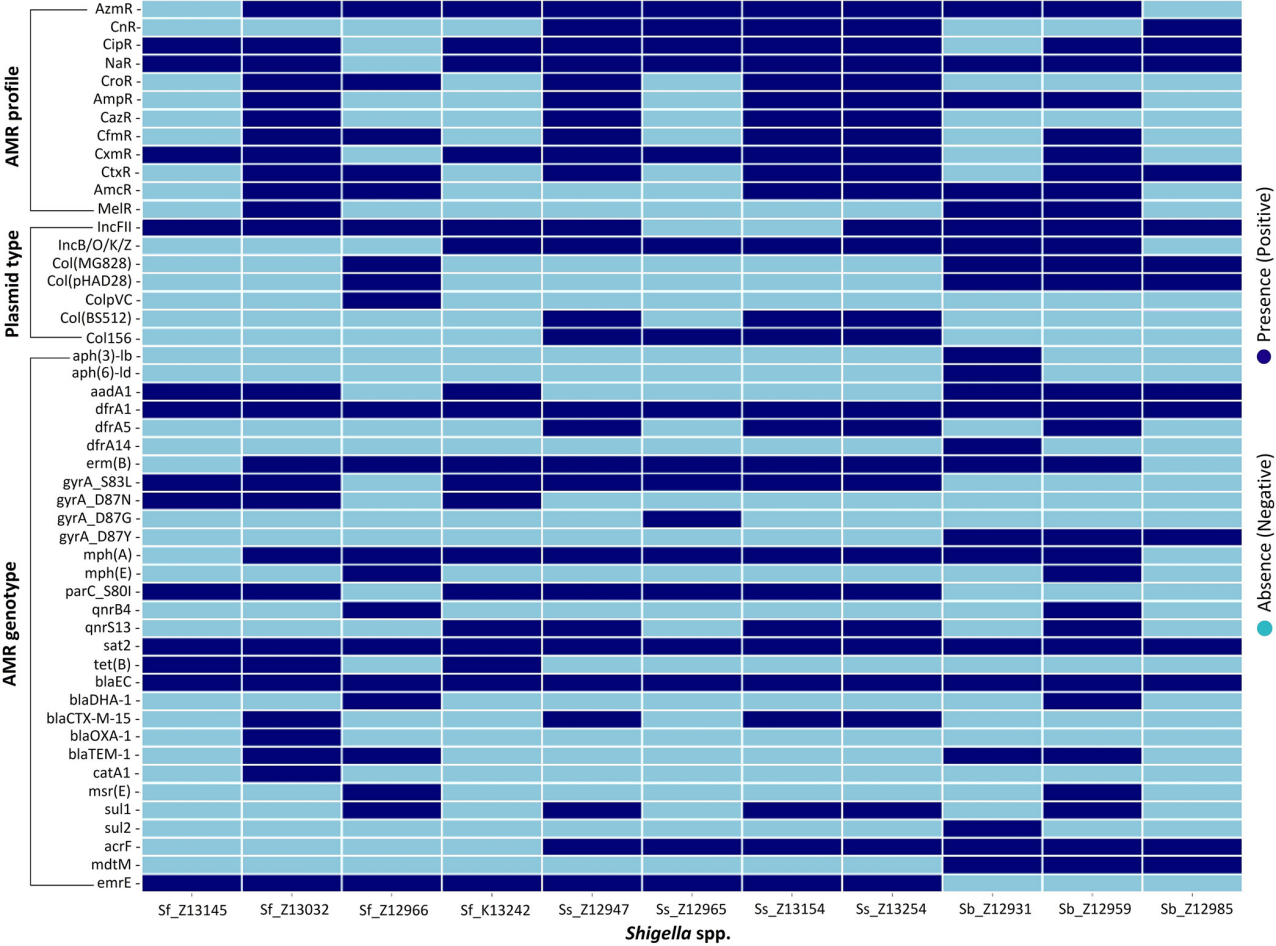
Heatmap of the AMR profile, plasmid type, and AMR genotype of 11 MDR *Shigella* strains. The *x* axis represents *Shigella* spp.; the *y* axis indicates the different AMR profiles, plasmid types, and AMR genotypes. Dark and light blue indicate the presence and absence of a specific aspect, respectively. R statistics v4.0.5 and the heatmaply package were used for the visualization (18, 19). R, resistant; Azm, azithromycin; Cn, gentamycin; Cip, ciprofloxacin; Na, nalidixic acid; Cro, ceftriaxone; Amp, ampicillin; Caz, ceftazidime; Cfm, cefixime; Cxm, cefuroxime; Ctx, cefotaxime; Amc, amoxicillin and clavulanate; Mel, amdinocillin; Sf, Shigella flexneri; Ss, Shigella sonnei; Sb, Shigella boydii; Sd, Shigella dysenteriae.

The resulting draft genome sequences contained 547 to 650 contigs, with sequencing depths between 30× and 70×. The PGAP annotation identified 4,778 to 5,223 coding sequences and 102 to 122 RNA genes (Table 1). Seven to sixteen AMR genes were identified in each isolate, substantiating the presence of multiple resistance mechanisms against different antimicrobials (Fig. 1). All isolates carried one or more types of plasmids (Fig. 1). However, the IncFII-type plasmid was the most common among 9 of 11 *Shigella* strains, which has been reported to cause intercontinental dissemination of multiple AMR factors and consequently drive *Shigella* endemics (4, 20). Therefore, the genomic data of these MDR *Shigella* spp. will facilitate our understanding of the inherent genetic mechanisms of AMR and contribute to AMR research, management, and surveillance.

**TABLE 1:** Assembly statistics and genome characteristics of 11 MDR *Shigella* spp. with respective accession numbers

Strain ID	rST^*a*^	GenBank assembly accession no.	Total length (bp)	GC content (%)	No. of contigs	Contig *N*_50_ (bp)	GenBank accession no.	Depth of sequencing^*b*^ (×)	Total no. of CDSs^*c*^	No. of RNA genes	SRA^*d*^ accession no.	Size (Mb)
Shigella flexneri												
Z13145	rST-1438	GCA_016806855.1	4,552,881	50.42	557	31,192	JAEUXK000000000	45	4,870	119	SRX10153410	295.3
Z13032	rST-1438	GCA_016858335.1	4,647,720	50.46	560	33,791	JAFDOL000000000	70	4,977	122	SRX10153411	481.5
Z12966	rST-15810	GCA_016774685.1	4,612,169	50.50	555	32,208	JAEUXL000000000	35	4,975	119	SRX10153413	264.3
K13242	rST-1438	GCA_016757315.1	4,651,679	50.47	568	33,791	JAESNI000000000	64	4,981	119	SRX10153323	288.2
Shigella sonnei												
Z12947	rST-1458	GCA_016858385.1	4,658,609	50.85	579	25,389	JAFDOM000000000	40	4,985	116	SRX10160715	275.7
Z12965	rST-1458	GCA_016858375.1	4,598,243	50.88	547	25,388	JAFDON000000000	45	4,881	122	SRX10160716	277.5
Z13154	rST-1458	GCA_016858345.1	4,636,612	50.89	561	25,389	JAFDOO000000000	45	4,953	116	SRX10160717	297.7
Z13254	rST-1458	GCA_016858475.1	4,826,932	50.67	650	25,009	JAFDOP000000000	45	5,223	118	SRX10160718	287
Shigella boydii												
Z12931	rST-15751	GCA_016820325.1	4,513,634	50.74	592	22,843	JAFBLF000000000	55	4,925	103	SRX10160639	358
Z12959	rST-15751	GCA_016888265.1	4,565,508	50.78	624	22,617	JAFEJL000000000	30	5,014	104	SRX10160637	211.7
Z12985	rST-15751	GCA_016888275.1	4,389,177	50.69	561	21,032	JAFEJM000000000	50	4,778	102	SRX10160638	415.9

a rST, ribosomal sequence type; rSTs were obtained from the rMLST database (http://pubmlst.org/rmlst/) (16).

b Sequence coverage was estimated using fastq-info v2.0 (https://github.com/raymondkiu/fastq-info) (17).

c CDSs, coding sequences.

d SRA, Sequence Read Archive.

### Data availability.

All data are available in GenBank under the BioProject accession numbers PRJNA693631, PRJNA694802, PRJNA698078, and PRJNA698772.
